# Antibody dynamics post-Comirnaty and CoronaVac vaccination in Malaysia

**DOI:** 10.1038/s41598-022-19776-3

**Published:** 2022-09-19

**Authors:** Cheng Siang Tan, Vaenessa Noni, Whilemena Upam Herman Ulok Melina, Ummi Syafiqah Abdorahman, Joseph Niler Bimbang, Natasya Marliana Abdul Malik, Mohammad Esa Hossen, Md Mizanur Rahman, Lela Su’ut, Asri Said, Claire Chen, Bego Sepop, Morni Abu Samat, John Chee Ming Chew, Dewi Mamora, Sian Kong Tan

**Affiliations:** 1 Faculty of Medicine and Health Sciences, Universiti Malaysia Sarawak, 94300 Kota Samarahan, Sarawak Malaysia; 2 Abbott Laboratories, 3 Frase Street #23-28 DUO Tower, Singapore, 189352 Singapore; 3 Advanced Pathology (M) Sdn Bhd, Kuching, 93400 Kuching, Sarawak Malaysia; 4 Klinik Morni, Uni Garden, 94300 Kota Samarahan, Sarawak Malaysia; 5 Borneo Medical Centre, 93350 Kuching, Sarawak Malaysia; 6 Normah Specialist Medical Centre, 93050 Petra Jaya, Kuching, Sarawak Malaysia

**Keywords:** Viral infection, RNA vaccines

## Abstract

Several vaccines have been fast-tracked through clinical trials to mitigate the progression of the SARS‑CoV‑2 pandemic. We analyzed sequential blood samples from 314 recipients of Comirnaty and CoronaVac in East Malaysia for the spike-binding IgG (IgG-S), nucleocapsid-binding IgG (IgG-N), spike-binding IgM (IgM-S) and serum vitamin D (VitD). A subset of samples was analyzed for the neutralizing antibodies (Ig-RBD). Results showed that IgG-S due to Comirnaty was significantly higher than CoronaVac. IgM-S was detected in 80.0% Comirnaty and 69.5% CoronaVac recipients, while IgG-N was detected in 58.1% CoronaVac but not in Comirnaty recipients. All IgG-S-positive vaccines possessed detectable Ig-RBD after the second dose but with a weak to moderate correlation. The serum VitD levels did not influence the antibody magnitude in both vaccines. In essence, SARS-CoV-2 vaccination is an IgG-S-dominant event, Comirnaty was more effective than CoronaVac in mounting IgG-S and Ig-RBD responses, independent of the patient’s VitD level.

## Introduction

### Background of the pandemic

The novel severe acute respiratory syndrome virus 2 (SARS-CoV-2) was first identified in Wuhan, China, at the end of 2019 and soon escalated to become a pandemic. As of November 2021, more than 262 million confirmed cases have been reported, with > 5.2 million deaths worldwide^1^. Two years on, we are still caught in the cyclical waxing and waning of SARS-CoV-2 infections that have crippled the economy and disrupted the healthcare system^2^. Moreover, although the Malaysian public health response effectively contained SARS-CoV-2 transmission in the first half of 2020, containment was lost due to the Sabah state election in the northern state of East Malaysia, which caused a surge in the community transmission rate nationwide^3^.

### Need for vaccines

Frantic international efforts have led to the development of several SARS-CoV-2 vaccine platforms, such as inactivated, live attenuated, recombinant protein, virus vectored, and mRNA vaccines^1^. As a result, three vaccines, namely, CoronaVac (Sinovac), Comirnaty (BNT162b2/Pfizer-BioNTech), and Adenovirus-vectored ChAdOx1 (Astrazeneca), were the first to be approved, and have been used widely in the Malaysia vaccination initiative. Notably, Sarawak, a state in East Malaysia, has one of the highest SARS-CoV-2 vaccination rates in the country, with a boasting coverage of more than 75% as of 3rd December 2021, and with CoronaVac being the most widely used (59.7%), followed by Comirnaty (38.5%) and ChAdOx1 (1.8%)^4^.

Both CoronaVac and Comirnaty were administered in two doses at three-week intervals, with published efficacy ranging from 94–95% to 50.7–83.5%, respectively^5–8^. However, the end-point of these observational studies was reported 14 d after the second dose, which did not consider the magnitude and durability of antibodies after the end-point.

### Principle of vaccination

Vaccination induces antibodies against the spike (S) glycoprotein of SARS-CoV-2. As a result, anti-S antibodies block the receptor-binding domain (RBD) on the S, preventing viral docking onto the angiotensin-converting enzyme 2 (ACE2) receptor, thereby averting infection. Although SARS-CoV-2 produces several structural proteins, such as the S, nucleocapsid (N), membrane (M), and envelope (E)^9^, antibodies against N, M, and E are not neutralizing in the absence of anti-S antibodies. Therefore, while Comirnaty delivers a nanolipid particle containing the mRNA coding for the whole S, CoronaVac uses an inactivated whole cultured SARS-CoV-2.

### Risk and protective factors

Studies have shown that patients with COVID-19, especially the elderly, obese, and male patients, including those with chronic diseases, such as hypertension, diabetes, chronic respiratory, and cardiovascular diseases, have a higher risk of COVID-19-related complications and mortality^10–12^. Besides, since hypovitaminosis D has been suggested as a risk factor, low serum vitamin D (VitD) correlates with poor prognosis among the COVID-19 patients^13–15^. Specifically, VitD is a fat-soluble hormone precursor that is synthesized in the skin upon exposure to the sun’s ultraviolet B (UVB) radiation and undergoes sequential hydroxylation to 25(OH)VitD and 1,25(OH_2_)VitD in the liver and kidney, respectively. Furthermore, since it has immunomodulatory functions, its deficiency has been associated with adverse outcomes in patients with respiratory tract infections, cardiovascular diseases, and autoimmune diseases^16,17^. Therefore, the VitD regulation of viral infections is inevitably more complex than initially thought. Nevertheless, the presence of vitamin D receptors on both T^18^ and B^19^ cells suggests that vitamin D plays a role in cytokine/chemokine regulation^20^ and viral clearance^21^. Hence, this prospective longitudinal study correlated the impact of serum VitD levels with the magnitude of antibodies produced post-Comirnaty and CoronaVac vaccinations.

## Results

### Subject characteristics

Blood samples were collected from 348 subjects following COVID-19 vaccinations. Among them, 46.3% (n = 161) were from Comirnaty, and 53.7% (n = 187) were from CoronaVac vaccine recipients. However, only 314 subjects (151 from Comirnaty and 163 from CoronaVac) were included in the analysis after excluding 34 subjects (10 from Comirnaty and 24 from CoronaVac) because of infection.

Table 1 illustrates the characteristics of the vaccine recipients by vaccine type. Analysis showed that although CoronaVac recipients were slightly younger (mean = 38.7, SD = 11.1 years) than Comirnaty recipients (Mean = 41.2, SD = 12.0), the difference between them was not statistically significant (*p* > 0.05). However, significant gender and ethnic differences were observed between Comirnaty and CoronaVac recipients, indicating a higher female-to-male ratio in the Comirnaty cohort than in the CoronaVac cohort (2.57 *vs.* 1.14). Additionally, while the Comirnaty cohort was high among the Natives (62.7%) and Foreigners (87.5%), CoronaVac was proportionately higher among the Chinese (63.3%). Investigations also revealed that of 314 vaccine recipients, 81(25.8%) had co-morbidities, such as diabetes mellitus, hypertension, dyslipidemia, and others, the co-morbidity pattern was equally distributed between both Comirnaty and CoronaVac recipients in general (*p* > 0.05), except for dyslipidemia, which was higher among the Comirnaty recipients (7.2%) than the CoronaVac recipients (1.8%) (*p* < 0.05).

**Table 1:** Characteristics of vaccinated recipients.

Characteristics	n	Vaccine type		*p* value
		Comirnaty	CoronaVac	
Age in years, mean (SD)^a^	313	41.2 (12.0)	38.7 (11.1)	0.058
**Gender (n = 315) (%)**^**b**^				
Female	195	108 (55.4)	87 (44.6)	< 0.001***
Male	118	42 (35.6)	76 (64.4)	
Female-to-male ratio	1.65	2.57	1.14	
**Ethnicity (n = 315) (%)**^**b**^				
Natives	147	85 (57.8)	62 (42.2)	< 0.001***
Chinese	158	58 (36.7)	100 (63.3)	
Foreigner	8	7 (87.5)	1 (12.5)	
Body mass index, mean (SD)^a^	314	26.8 (5.2)	25.7 (4.9)	0.058
**Co-morbidity**^**b**^				
Diabetes mellitus	13	3.9	4.3	0.887
Hypertension	38	12.4	11.7	0.801
Dyslipidemia	14	7.2	1.8	0.020*
Others (asthma, eczema, etc.)	36	12.4	10.4	0.550

^a^*p* value was set according to the independent sample t-test.

^b^*p* value was obtained from the chi-square test.

*p < 0.05; **p < 0.01; ***p < 0.001.

### Serum IgG-S binding antibody titer

None of the included participants tested positive for the IgG-S antibody (relative to the manufacturer’s cutoff at 50 AU/mL) at week zero (pre-vaccination) (Table 2). However, at week three, 97.9% and 54% of Comirnaty and CoronaVac recipients developed positive IgG-S antibody responses (≥ 50 AU/mL). Specifically, while all Comirnaty recipients mounted positive IgG-S antibodies three weeks and 13 weeks (week six and 16) after the second dose, 98.1% and 97.1% of CoronaVac recipients mounted a positive antibody response at week six and week 16, respectively.

**Table 2:** Qualitative antibody responses to the Comirnaty and CoronaVac recipients.

Follow-up	IgG-S				IgG-N				IgM-S			
	Comirnaty		CoronaVac		Comirnaty		CoronaVac		Comirnaty		CoronaVac	
	n	%	n	%	n	%	n	%	n	%	n	%
W-0	0/148	0.0	0/163	0.0	0/148	0.0	0/163	0.0	0/148	0.0	0/163	0.0
W-3	139/142	97.9	86/158	54.4	0/142	0.0	0/158	0.0	81/142	57.0	31/158	19.6
W-6	135/135	100.0	151/154	98.1	0/135	0.0	90/154	58.4	108/135	80.0	107/154	69.5
W-16	136/136	100.0	136/140	97.1	0/136	0.0	3/140	2.1	13/136	9.6	9/140	6.4

*Seropositivity thresholds: IgG-S (≥ 50 AU/mL), IgG-N (≥ 1.4 S/C), and IgM-S (≥ 1.0 S/C) are used.

W-0, Week zero; W-3, Week three; W-6, Week six; W-16, Week 16.

Non-parametric Mann–Whitney U test indicated a statistically significant difference in the IgG-S antibody titers between the Comirnaty and CoronaVac recipients (*p* < 0.001), the antibody titer was significantly higher among the Comirnaty than in CoronaVac recipients (Table 3).

**Table 3:** Median IgG-S antibody titers over time, based on the vaccine type.

Type of vaccine	n	IgG-S [median (IQR)]						
		W-0	n	W-3	n	W-6	n	W-16
Comirnaty	148	3.00 (0.25, 7.00)	142	1307.50 (734.25, 2777.00)	135	14,113.00 (8035.00, 23,113.00)	136	2603.50 (1669.00, 4175.75)
CoronaVac	163	1.00 (0.00, 3.00)	158	66.00 (29.00, 133.00)	154	949.50 (554.75, 1539.00)	140	214.50 (132.50, 377.75)
*p* value		N/A		< 0.001***		< 0.001***		< 0.001***

Samples (n) and interquartile ranges (IQRs) indicate the minimum and maximum values.

*p* value was set according to the Mann–Whitney U test.

W-0, Week zero; W-3, Week three; W-6, Week six; W-16, Week 16.

*p < 0.05; **p < 0.01; ***p < 0.001.

**Figure 1:**
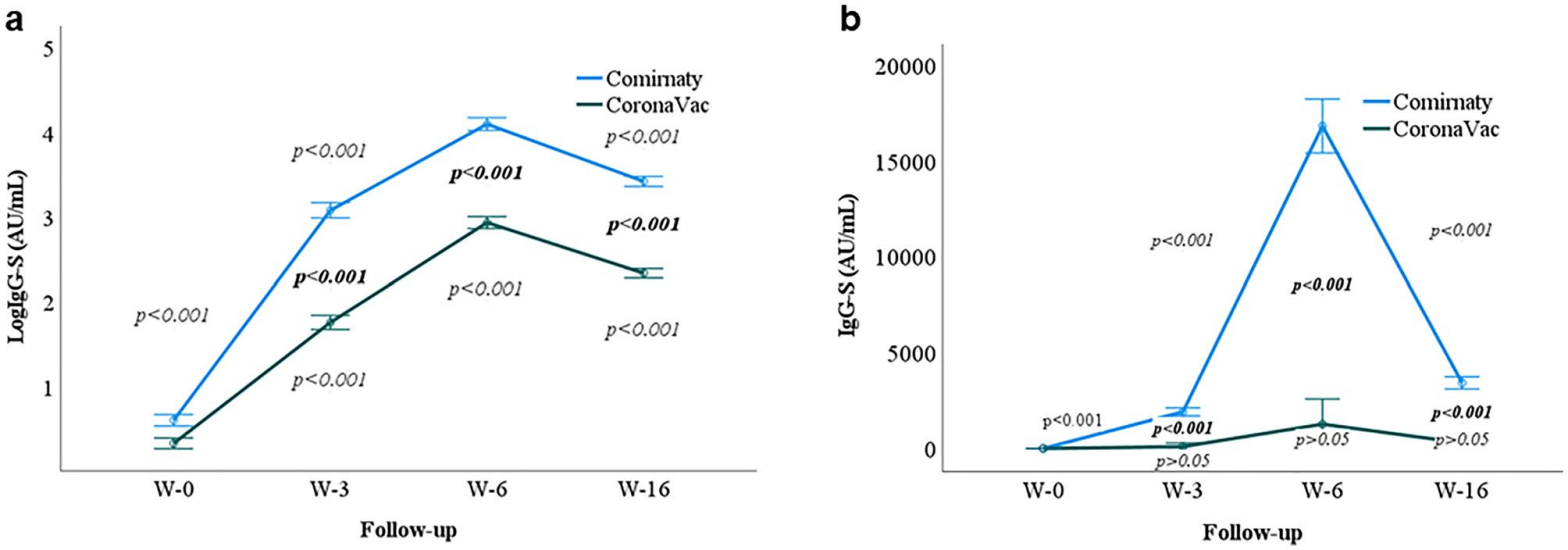
The IgG-S titer of Comirnaty and CoronaVac vaccine recipients at weeks 0, 3, 6, and 16. (**a**) Geometric mean IgG-S titer, (**b**) mean IgG-S titer. Black dotted and dashed lines represent the manufacturer’s medium and high positive cutoff values. W-0, Week zero; W-3, Week three; W-6, Week six, W-16, Week 16. Regular *p* value indicating the difference between two sampling points. Bolded *p* values indicating the difference between Comirnaty and CoronaVac at the respective week.

Furthermore, of the 314 understudied subjects, 256 had complete IgG-S antibody values for all sampling points. After removing the outliers, data from 240 subjects were analyzed with repeated measures ANOVA. Data analysis revealed that the antibody IgG-S significantly increased from week zero to 16 observations in Comirnaty and CoronaVac recipients (*p* < 0.001). However, the pair-wise analysis between Comirnaty and CoronaVac vaccines indicated that the IgG-S titer was significantly higher among the Comirnaty recipients than that of the CoronaVac recipients at weeks 3, 6, and 16 (*p* < 0.001) (Fig. 1a).

Additionally, the binding IgG-S assay (untransformed) analysis showed that the antibody magnitude due to Comirnaty was consistently higher than that of CoronaVac. Results showed that while Cominarty recipients achieved a positively medium and high range between weeks three and six, respectively, they waned to a positive medium value after week six. However, the mean antibody titer due to CoronaVac remained positively low throughout weeks three, six, and 16 (Fig. 1b).

### Serum Ig-RBD neutralizing antibody titer

**Figure 2:**
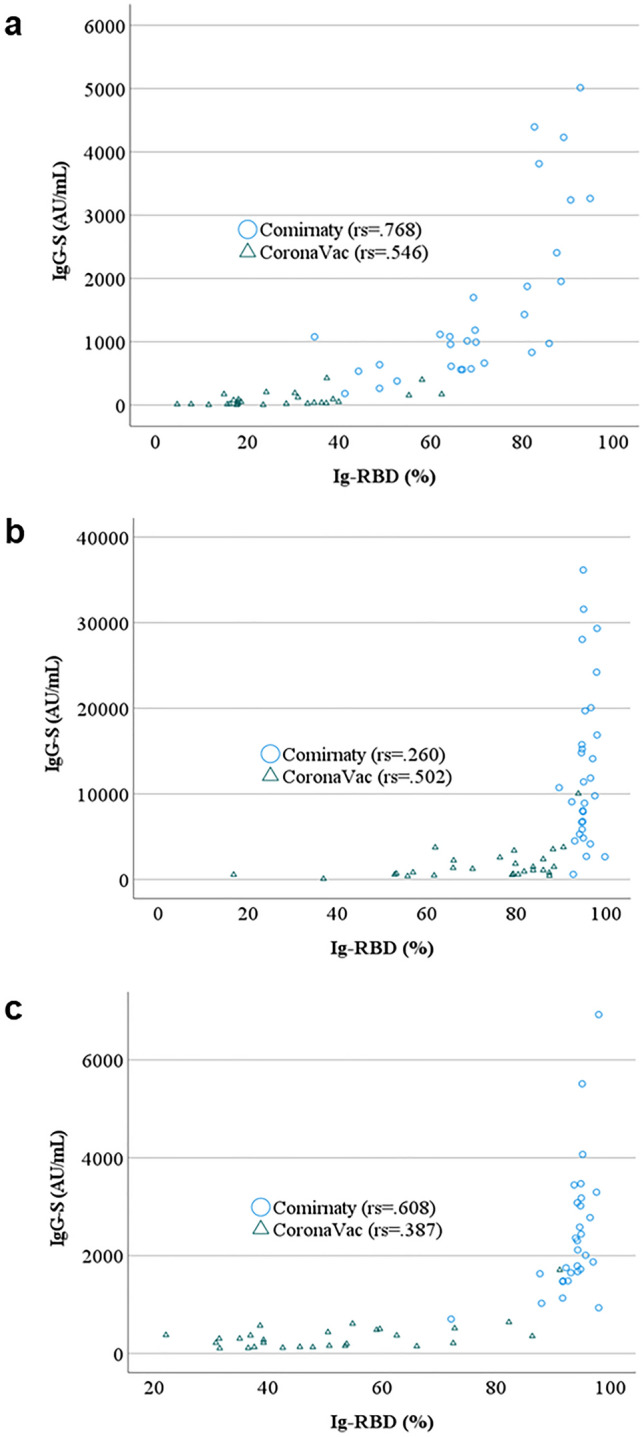
Correlation between IgG-S and Ig-RBD of Comirnaty (n = 30) and CoronaVac (n = 28) recipients. IgG-S was determined using SARS-CoV-2 IgG Quant II (Abbott) and Ig-RBD determined using the cPASS surrogate virus neutralization test (Genscript). Spearman rank-order correlation coefficient is represented as rs. (**a**) Week 3, (**b**) Week 6 and (**c**) Week 16.

Subsequently, the neutralizing antibody (Ig-RBD) titer measured (%inhibition) was assayed in a subset of Comirnaty (n = 30) and CoronaVac (n = 28) recipients. We observed that all Comirnaty recipients produced neutralizing antibodies above the manufacturer’s positive cutoff (30% inhibition at week 3; mean = 71% inhibition) and increased to 95% at week 6 before declining slightly to 93% inhibition at week 16. However, contrary to Comirnaty recipients, although no neutralizing antibodies were detected after the first dose of CoronaVac at week three, antibodies were only detectable at 72% inhibition after week six. Still, this value declined steeply to 51% inhibition at week 16. Spearman rank-order correlation analysis indicated that although IgG- correlated positively with all periods with S Ig-RBD, the associations are not strong (Fig. 2a–c). Comirnaty and CoronaVac have the weakest correlation at Week 6 (*rs* = 0.260) (Fig. 2b) and Week 16 (*rs* = 0.387) (Fig. 2c) respectively.

### Serum IgG-N antibody titer

No Comirnaty recipient exhibited positive IgG-N responses throughout the 16 weeks of observation (Table 2). However, for the CoronaVac recipients, while IgG-N was undetectable at weeks zero and three (three weeks after the first dose), it was detected in 58.4% of the participants at week six (three weeks after the second dose) and declined to 2.1% at week 16.

Additionally, the nonparametric Mann–Whitney U test revealed that while the serum IgG-N antibody level was significantly higher among the CoronaVac vaccine recipients than the Comirnaty recipients, the CoronaVac recipients showed consistently higher antibody titers than the Comirnaty vaccine recipients. Table 4 illustrates the median with interquartile serum ranges of IgG-N antibodies over time.

**Table 4:** Serum IgG-N antibody titers over time, based on the vaccine recipients.

Type of vaccine	n	Serum IgG [median S/C (IQR)]						
		W-0	n	W-3	n	W-6	n	W-16
Comirnaty	148	0.03 (0.02, 0.05)	142	0.04 (0.02, 0.08)	135	0.03 (0.02, 0.07)	136	0.03 (0.02, 0.07)
CoronaVac	163	0.04 (0.03, 0.06)	158	0.08 (0.05, 0.16)	154	1.04 (0.47, 2.00)	140	0.25 (0.13, 0.47)
*p* value		N/A		< 0.001***		< 0.001***		< 0.001***

Samples (n), Signal/cutoff (S/C), and interquartile ranges (IQRs) indicate the minimum and maximum values.

*p* value was set according to the Mann–Whitney U test.

W-0, Week zero; W-3, Week three; W-6, Week six; W-16, Week 16.

*p < 0.05; **p < 0.01; ***p < 0.001.

**Figure 3:**
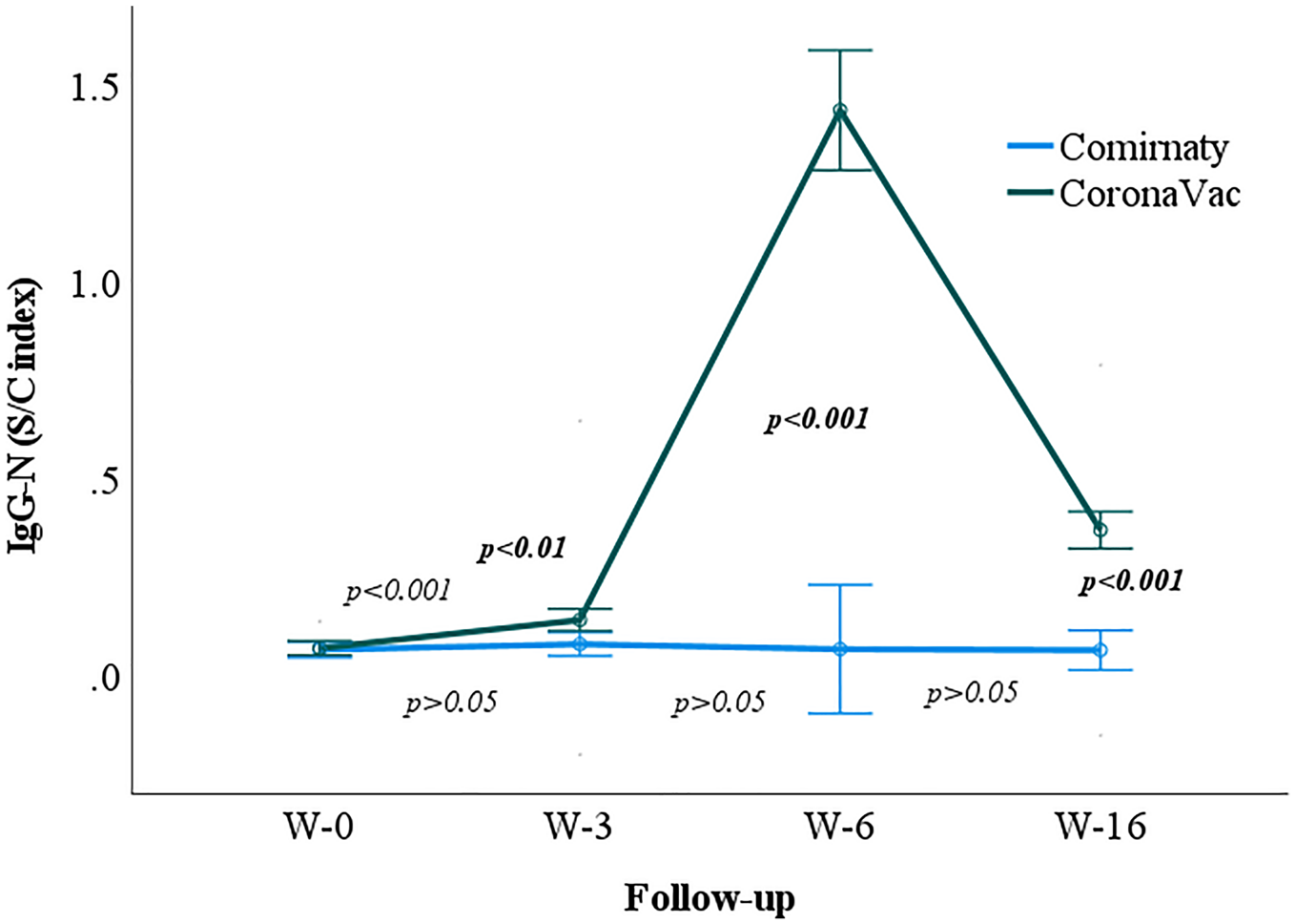
Geometric mean of the IgG-N S/C index in Comirnaty and CoronaVac vaccine recipients at weeks 0, 3, 6, and 16. W-0, Week zero; W-3, Week three; W-6, Week six, W-16, Week 16. Regular *p* value indicating the difference between two sampling points. Bolded *p* values indicating the difference between Comirnaty and CoronaVac at the respective week.

Data analysis also revealed that although the IgG-N antibody titers remained statistically unchanged throughout the observation points (*p* > 0.05) among the Comirnaty recipients, the antibody IgG-N increased significantly from week zero to six, followed by a decrease at week 16 (*p* < 0.05) among the CoronaVac recipients. Besides, the pair-wise analysis between Comirnaty and CoronaVac recipients indicated no statistically significant mean difference between Comirnaty and CoronaVac recipients at week zero (*p* > 0.05). Nevertheless, a significant mean difference was observed between Comirnaty and CoronaVac recipients from weeks three to 16 (*p* < 0.001) (Fig. 3).

### Serum IgM-S antibody titer

This study analyzed 311 (Comirnaty = 148, CoronaVac = 163) subjects for the IgM-S antibody. Although the nonparametric Mann–Whitney U test indicated no statistically significant difference between the Comirnaty and CoronaVac IgM-S antibody titer at week zero and 16 (*p* > 0.05), a statistically significant difference was observed between weeks three and six (*p* < 0.05), indicating that the antibody titer was higher among the Comirnaty recipients than the CoronaVac recipients (Table 5). Investigations also revealed that while 0% and 19.6% of Comirnaty and CoronaVac recipients mounted positive IgM-S responses three weeks after the first dose, the proportion increased to 80% (Comirnaty) and 69.5% (CoronaVac) at week six before decreasing to 9.6% (Comirnaty) and 6.4% (CoronaVac) at week 16 (Table 2).

**Table 5:** Serum IgM-S antibody titers over time, based on the vaccine recipients.

Type of vaccine	n	Serum IgM-S [median S/C (IQR)]						
		W-0	n	W-3	n	W-6	n	W-16
Comirnaty	148	0.04 (0.03, 0.07)	142	1.24 (0.50, 2.85)	135	2.17 (1.09, 4.29)	135	0.23 (0.12, 0.43)
CoronaVac	163	0.05 (0.03, 0.07)	158	0.38 (0.20, 0.76)	154	1.88 (0.84, 3.27)	140	0.21 (0.11, 0.50)
*p*-value		N/A		< 0.001***		< 0.022*		0.430

Samples (n), Signal/cutoff (S/C), and interquartile ranges (IQRs) indicate the minimum and maximum values.

W-0, Week zero; W-3, Week three; W-6, Week six, W-16, Week 16.

*p* value was set according to the Mann–Whitney U test.

*p < 0.05; **p < 0.01; ***p < 0.001.

**Figure 4:**
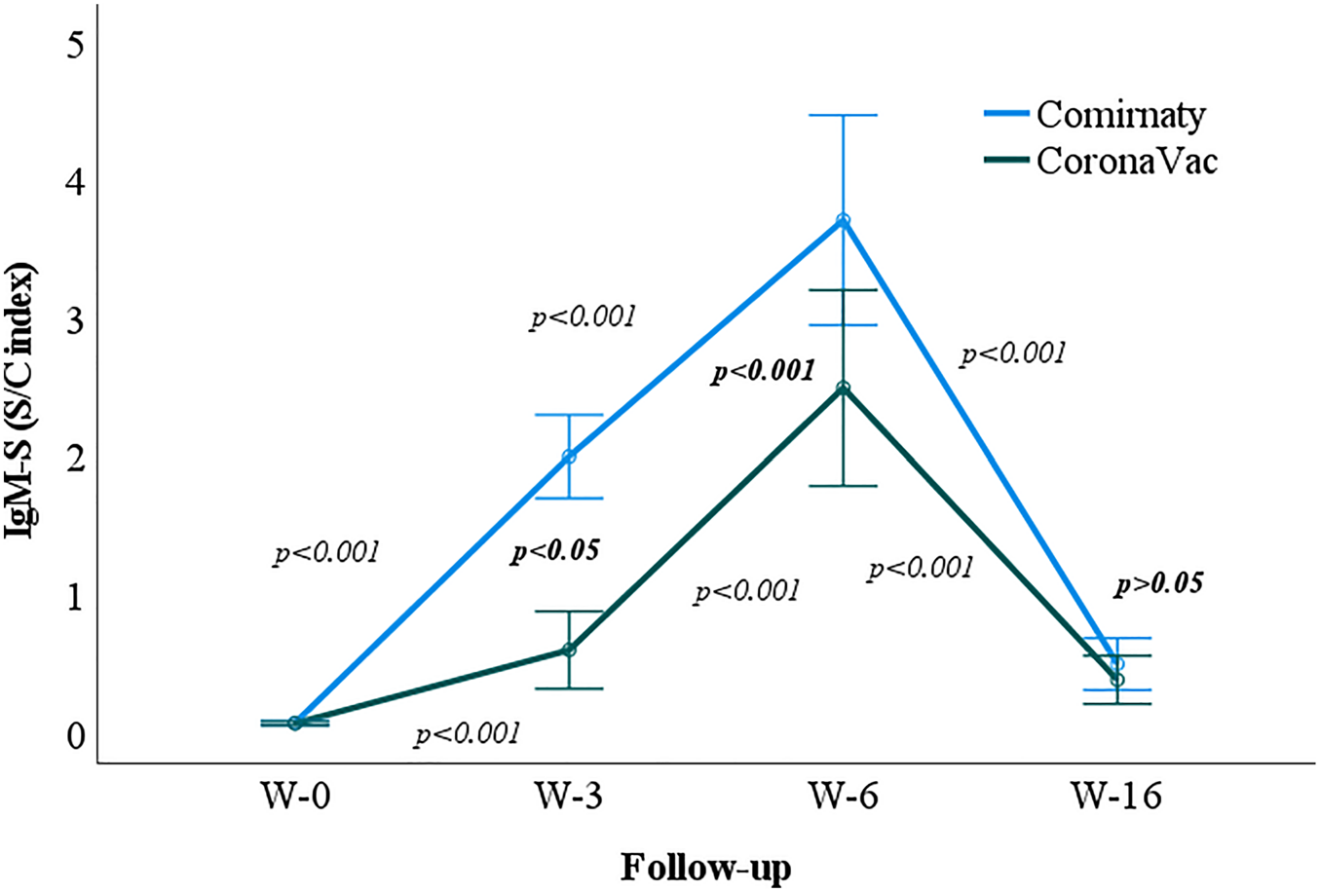
Geometric mean of the IgM-S S/C index in Comirnaty and CoronaVac vaccine recipients at weeks 0, 3, 6, and 16. W-0, Week zero; W-3, Week three; W-6, Week six; W-16, Week 16. Regular *p* value indicating the difference between two sampling points. Bolded *p* values indicating the difference between Comirnaty and CoronaVac at the respective week.

Subsequently, after removing the multivariate outliers (31 outliers), data of 225 subjects were analyzed. Repeated measures ANOVA analysis indicated that the IgM antibody level significantly increased from week zero to six, irrespective of the vaccine type. It also significantly decreased at week 16 in both Comirnaty and CoronaVac vaccines (*p* < 0.001). Contrastively, the pair-wise analysis showed that no statistically significant mean difference was observed between the Comirnaty and CoronaVac in different follow-ups (*p* > 0.05), except at week three (*p* < 0.001), where Comirnaty vaccine recipients showed a higher level of antibody (mean = 1.499) than CoronaVac (mean = 0.581) (Fig. 4).

### VitD and antibody kinetics

**Figure 5:**
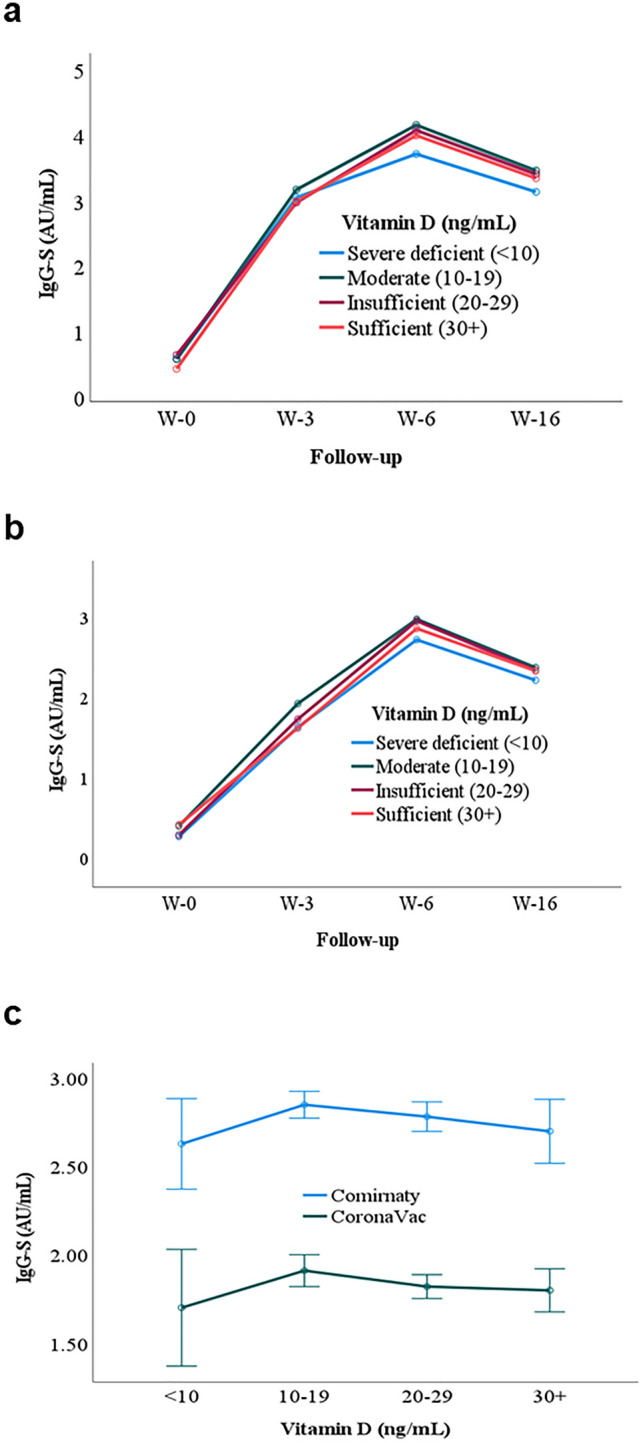
VitD and antibody kinetics. (**a**) Comirnaty, (**b**) CoronaVac. Graphs show the geometric mean IgG-S values of different VitD levels against the follow-up. (**c**) Comparison between the geometric mean of IgG-S resulting from Comirnaty and CoronaVac against the VitD level. W-0, Week zero; W-3, Week three; W-6, Week six, W-16, Week 16.

The geometric IgG-S mean titer of Comirnaty (Fig. 5a) and CoronaVac (Fig. 5b) groups were plotted against different serum VitD levels, namely, severe deficiency, deficiency, insufficient, and sufficient. Repeated measures ANCOVA (analysis of covariance) analysis indicated no significant association between serum VitD levels and the magnitude of antibody levels in both Comirnaty and CoronaVac recipients (Fig. 5c).

We also observed that while subjects’ VitD levels measured at weeks zero, three, and six were consistent and insignificantly different, 13.4% of these subjects had sufficient serum VitD levels, whereas 43.3%, 40.4%, and 2.9% had insufficient, moderate deficient and severe deficiency, respectively (Table 6).

**Table 6:** Classification of the VitD status.

Type of vaccine	Classification of VitD status (ng/mL)				Total
	Severe deficiency* (< 10)	Moderate deficiency* (10–19)	Insufficiency* (20–29)	Sufficiency* (30+)	
Comirnaty (n/%)	5 (55.6)	80 (63.0)	52 (38.2)	14 (33.3)	151 (48.1)
CoronaVac (n/%)	4 (44.4)	47 (37.0)	84 (61.8)	28 (66.7)	163 (51.9)
Total N (%)	9 (2.9)	127 (40.4)	136 (43.3)	42 (13.4)	314 (100)

W-0, Week zero; W-3, Week three; W-6, Week six; W-16, Week 16.

*Classification of Vitamin D deficiency^22^.

## Materials and methods

### Study design

We conducted this prospective longitudinal cohort study in Sarawak, Malaysia. Recipients of Comirnaty and CoronaVac were invited to participate in the study. Before inclusion, participants were given time to go through the participant information sheet, after which they signed an informed consent form agreeing with sampling and using their data for research and publication. Then, peripheral blood was taken at week zero (immediately before the first dose), week three (immediately before second dose), week six (three weeks post second dose), and week 16 (13 weeks post second dose).

The inclusion criteria included patients 18 years or older with no confirmed SARS-CoV-2 infection via molecular assay before receiving the first vaccine dose. However, participants with serologic evidence of natural infections at week zero or a breakout of infection during the study were excluded from the analysis. Subsequently, participants were notified of their test results.

### Chemiluminescence microparticle immunoassay

The SARS-CoV-2 Spike-binding IgG (IgG-S), nucleocapsid-binding IgG (IgG-N), and Spike-binding IgM (IgM-S) were measured using chemiluminescence microparticle immunoassays (CMIA), namely, the SARS-CoV-2 IgG II Quant, the SARS-CoV-2 IgG assay, and the SARS-CoV-2 IgM assay using the ARCHITECT i1000SR Analyzer (Abbott Laboratories, IL, US). Subsequently, serum 25-hydroxyvitamin (25-OH) VitD levels were measured using the 25-OH Vitamin D assay on the same analyzer.

### Neutralizing antibody assay

Neutralizing antibodies (Ig-RBD) targeting the receptor-binding domain of SARS-CoV-2 were measured using the cSARS-CoV-2 Surrogate Virus Neutralization Test Kit (Genscript, NJ, USA).

### Data analysis

Data analysis was performed using the IBM SPSS v.27. Then, the quantitative variables were presented as mean, standard deviation, and median with an interquartile range. The previously infected cases were excluded from the final analysis, as identified by positive IgG-S, IgG-N, or IgM-S values at week 0. Subsequently, the nonparametric Mann–Whitney U test was used to determine differences between Comirnaty and CoronaVac recipients. Next, to determine the effectiveness of Comirnaty and CoronaVac and to assess the impact of VitD on antibody titers, repeated measures ANCOVA were performed in which the base VitD was the covariate. However, the model also entered other variables to determine their impact. Finally, before interpreting the results, multivariate outliers were determined using the Mahalanobis distance and studentized residuals, after which the outlier cases were removed from the final analysis and checked using a repeated measure ANCOVA assumptions. A *p*-value of less than 0.05 was considered statistically significant. The correlation between IgG-S and Ig-RBD was analysed using Spearman rank-order correlation.

### Ethics declarations

This study was approved by the Universiti Malaysia Sarawak Medical Research Ethics Committee (Ref: ETIKA FME21-55).

## Discussion

### Binding and neutralizing antibodies

The SARS-CoV-2 vaccination is an effective way to mitigate the progression of the COVID-19 pandemic. Based on this fact, fully vaccinated individuals are reportedly 42.2 times (Comirnaty) and 12.5 times (CoronaVac) less likely to die compared to the unvaccinated individuals^23^. Approximately 78.6% of the Malaysian population had been fully vaccinated as of 11th January 2022. As reported, although the ICU admission rate for those who had completed their vaccination was 0.0066% (n = 954/14,599,984), the death rate was 0.01% (n = 1445/14,599,984). Moreover, national statistics showed that CoronaVac recipients were 5.5 and 3 times more likely to require ICU care and die compared with the Comirnaty recipients^24,25^. This result was not surprising since the antibody levels elicited by Comirnaty at weeks three, six, and 16 were 1.76-, 1.39- and 1.46-fold higher, respectively, than those from CoronaVac. Additionally, while higher antibody titers were reportedly correlated with better protection against severe COVID-19 and death^26,27^, the antibody levels waned 1.2- and 1.25-fold from weeks six to 16, suggesting that protection following vaccination decreased with time^28^, a trend that agrees with the observations by others in Hong Kong and Chile^27,29,30^. Other studies focusing solely on the antibody kinetics due to Cominarty, such as in Israel^31,32^, Estonia^33^, Hungary^33^, Finland^34^, and Greece^35^, reported similar waxing and waning patterns of IgG-S at comparable time points. Our study also showed that all Comirnaty recipients seroconverted with IgG-S antibody using the assay provider’s cutoff established at 50 AU/mL (seropositivity threshold) and remained positive at week 16. However, 1.9% of CoronaVac recipients failed to seroconvert three weeks after the second dose, with 2.9% having an antibody level of < 50 AU/mL at week 16. In a previous study, 1.9% of the nonresponders were immunocompetent without co-morbidities, which was interesting since nonresponders are usually seen in immunocompromised patients, those with psoriatic arthritis on methotrexate treatment, and those with chronic lymphocytic leukemia^27^. This finding extrapolates that approximately 2 in every 100 CoronaVac recipients will fail to seroconvert after the second dose, and 3 in 100 will not have positive IgG-S four months after initiating CoronaVac vaccination. Additionally, another study proposed that while 137,254 (n = 7223,897) fully vaccinated CoronaVac individuals failed to produce IgG-S, 209,493 (n = 7223,897) did not have positive IgG-S at week 16^24^. The seroconversion failure rate for CoronaVac in other studies was 2.3%-3.74% (Turkey)^36,37^ and 0.6% (Indonesia)^38^.

Alternatively, although the neutralizing antibodies elicited by complete doses of Comirnaty were significantly higher than those in CoronaVac at all periods, similar to IgG-S, the IgG-S levels of Comirnaty and CoronaVac cohorts were positively correlated with Ig-RBD. Still, the correlation was not as high as was reported by Levin and co-workers^32^ in their Comirnaty cohort. Nevertheless, to an extreme, Muller and co-workers reported that only 68.7% of their cohorts who were positive for IgG-S by ELISA were also positive for Ig-RBD^39^, implying that not all S-specific antibodies targeted the SARS-CoV-2 RBD.

By the end of December 2021, the regulatory body in Malaysia had recommended that the time interval for the booster dose vaccination should be as early as three months after receiving the respective primary vaccine, which agrees with the recommendation in the UK, Australia, and Canada^40^. However, although our findings support the three-month booster interval recommendation for CoronaVac, it disagrees with Comirnaty, which we believe can span over a longer time interval due to the significantly higher IgG-S level of Comirnaty compared with CoronaVac at week 16.

### Qualitative IgG-N and IgM-S

We report that all Comirnaty recipients had no detectable IgG-N as expected. Meanwhile, no CoronaVac recipient had detectable IgG-N after the first dose, the positivity increased to 58.4% three weeks after the second dose (week six), which is similar to the findings of others in Thailand and Southern Brazil, whereby 1%^41^ and 57–62.8%^41,42^ were IgG-N positive after the first and second doses, respectively. Interestingly, Muena et al., reported that CoronaVac failed to boost the IgG-N titer in previously infected individuals, concluding that CoronaVac is a poor N immunogen^29^. This finding makes IgG-N a suboptimal biomarker for seroprevalence and antibody kinetics studies^43^. Furthermore, it has been estimated that the half-life of IgG-S is double that of IgG-N^44^. However, only 85.7% of SARS-CoV-2-infected patients in a previous study were seropositive for IgG-N 30 days after the infection, with seropositivity declining by 38.6%, 23.7%, and 26% at 6, 9, and 12 months, respectively^45^.

Our investigations also revealed that not all Comirnaty and CoronaVac recipients mounted an IgM-S response, with only 80% and 69%, respectively, producing a seropositive response after the second dose. However, this figure declines rapidly to 9.6% (Comirnaty) and 6.4% (CoronaVac), respectively, at week 16. Nevertheless, even though not all natural SARS-CoV-2 infections mounted an IgM response, the presence of IgM was associated with shorter virus shedding^46^, suggesting that SARS-CoV-2 vaccination and infection mainly mount IgG-S response^47^. Therefore, diagnostic tools for detecting IgG-N and IgM-S may be suboptimal, resulting in false negatives.

### VitD level and antibody kinetics

We observed that the VitD level was not a determinant of the antibody magnitude in both Comirnaty and CoronaVac recipients, discounting the hypothesis that higher VitD levels may boost the magnitude of IgG-S titers, thereby conferring better protection. Our observation agrees with a German study whereby recipients of BNT162b2 (Comirnaty), regardless of VitD supplementation status, did not exhibit any significant difference in antibody magnitude^48^. Therefore, their findings suggest that the VitD effect on antibody magnitude was not affected by vaccine type (mRNA and whole inactivated) or race. Our observation also agrees with pneumococcal vaccination, in which the authors observed no correlation between VitD level and the magnitude of anti-*Streptococcal pneumoniae* antibody levels^49^. Alternatively, studies on human papillomavirus (HPV), rubella, and measles vaccinations have demonstrated that VitD levels were inversely proportional to lower antibody titers^50–52^. However, these findings were inconsistent with the observations that lower VitD levels were associated with a poorer prognosis in COVID-19 and other respiratory tract infections^17,21,53,54^. One feasible explanation is that higher VitD levels may similarly inhibit the production of antibodies to the downregulation of pro-inflammatory cytokines^55^. Still, this hypothesis is not in sync with our findings.

Interestingly, we discovered that while 43.3% of our subjects were suffering from hypovitaminosis D, defined as having a mean serum VitD level < 20 ng/mL^56^, 2.9% of them were suffering from severe hypovitaminosis D (< 10 ng/mL)^22^. In contrast, only 13.4% had sufficient VitD (> 30 ng/mL). This finding contradicts the perception that people living near the equator would receive ample and consistent sunlight throughout the year, which should synthesize VitD on the skin^57^. Notably, our participants have been subjected to nationwide lockdown since 18th March 2020 and spent most of their time indoors^2^, which may have contributed to the high prevalence of hypovitaminosis D among the study population. Our study is the first to report the prevalence of hypovitaminosis D in East Malaysia. Compared to pre-pandemic studies in West Malaysia, hypovitaminosis D is relatively common, with the prevalence ranging from 42.6 to 70%^58–61^. Therefore, this observation should be taken seriously since numerous studies have demonstrated that hypovitaminosis D correlates with a higher risk of developing severe COVID-19 and even death^57,62^, suggesting that prolonged lockdown without VitD supplementation may predispose nearly half of the population to a higher risk of severe COVID-19 upon infection, including other deficiency-related diseases, such as diabetes, multiple sclerosis, rheumatoid arthritis, tuberculosis, sepsis, respiratory diseases, dementia and Alzheimer, cardiovascular diseases, and Rickett and osteoporosis^16,63,64^.

## Limitations

First, our study was based on the measurement of binding antibody titers and a subset of sequential samples was tested using the surrogate virus neutralization test (sVNT). However, the gold-standard plaque reduction neutralization test (PRNT_50_) was not conducted. Second, the T-cell response was not evaluated, which may be crucial in inferring vaccine protection. Third, since a serum VitD prevalence study had never been conducted in East Malaysia before the pandemic, we could not ascertain the impact of the prolonged lockdown on serum VitD levels. Moreover, since the VitD supplementation history was not collected for this study, we could not exclude the possible higher level of VitD in VitD-supplemented subjects.

## Conclusion

Serum VitD levels do not significantly affect the magnitude of IgG-S resulting from Comirnaty or CoronaVac vaccinations. Moreover, Comirnaty mounted a stronger humoral response and produced a longer-lasting IgG-S than CoronaVac. However, not all vaccinated subjects mounted an IgG-N and IgM-S response.

## Data Availability

Anonymised data used in this manuscript may made available from the corresponding author upon reasonable request.
